# Cancer registries in four provinces in Turkey: a case study

**DOI:** 10.1186/1744-8603-8-34

**Published:** 2012-10-30

**Authors:** Frances A Stillman, Michelle R Kaufman, Naseeb Kibria, Sultan Eser, Mark Spires, Yusuf Pustu

**Affiliations:** 1 Johns Hopkins Bloomberg School of Public Health, Baltimore, MD, USA; 2 Izmir Cancer Registry, Izmir, Turkey; 3 New Hope in Health Foundation (SUVAK), Ankara, Turkey

**Keywords:** Cancer surveillance, Turkey, Case study, International collaboration, Cancer registry

## Abstract

**Background:**

The burden of cancer affects all countries; while high-income countries have the capacity and resources to establish comprehensive cancer control programs, low and middle-income countries have limited resources to develop such programs. This paper examines factors associated with the development of cancer registries in four provinces in Turkey. It looks at the progress made by these registries, the challenges they faced, and the lessons learned. Other countries with similar resources can benefit from the lessons identified in this case study.

**Methods:**

A mix of qualitative case study methods including key informant interviews, document review and questionnaires was used.

**Results:**

This case study showed that surveillance systems that accurately report current cancer-related data are essential components of a country’s comprehensive cancer control program. At the initial stages, Turkey established one cancer registry with international support, which was used as a model for other registries. The Ministry of Health recognized the value of the registry data and its contribution to the country’s cancer control program and is supporting sustainability of these registries as a result.

**Conclusions:**

This study demonstrates how Turkey was able to use resources from multiple sources to enhance its population based cancer registry system in four provinces. With renewed international interest in non-communicable diseases and cancer following the 2011 UN high-level meeting on NCDs, low- and middle- income countries can benefit from Turkey’s experience. Other countries can utilize lessons learned from Turkey as they address cancer burden and establish their own registries.

## Background

The global burden of cancer has increased over the years, resulting in 7.6 million deaths in 2008, with an estimated two-thirds in low- and middle-income countries (LMICs) [1]. At this rate, an estimated 13 million deaths are expected to result from cancer by 2030. Although cancer is associated with more deaths than HIV/AIDS in resource-poor countries [2], it is frequently classified into a broader category with other diseases in many of these countries, as well as on the international community’s health care agenda [3-6]. Although cancer control efforts seem to be obscure in the current global health agenda, in 2005 the World Health Organization (WHO) passed the *Resolution on Cancer Control WHA 58.22*, urging Member States to conduct cancer control and prevention activities [7,8]. A key provision in the *Resolution* was to develop a comprehensive national cancer control programme and integrate it into a country’s existing health care system. The WHO resolution also states that all countries committed to alleviating the burden of cancer, regardless of their resource level, should aim to implement a surveillance system [9].

### Cancer registries in low to middle income countries

“Cancer surveillance is the ongoing, timely, and systematic collection and analysis of information on new cancer cases, extent of disease, screening tests, treatment, survival, and cancer deaths” [10]. Surveillance systems, including cancer registries, allow countries to obtain specific data by geographic region on people diagnosed with cancer and to use the data to develop preventive, diagnostic, or therapeutic practices; to assess the efficacy of these interventions and initiate research studies; and to develop policies and allocate funding [10-12]. Cancer registries that are set up throughout a country to collect and record data and report cancer cases in the areas they cover are the foundation of cancer surveillance activities. However, it has been acknowledged that there is a “significant lack of relevant cancer data from developing countries” [12,13]. Registries require time, effort, expertise and skills development so they can become functional and meet international standards. LMIC often lack cancer registries or “the cancer registration data may be of low quality” [13]. Thus, it is important to gain a better understanding of the capacity and factors that could help develop and improve country cancer registries. In trying to establish cancer registries, LMICs often struggle with inadequate health services, transient populations, lack of funding and of trained personnel, and incomplete or inaccurate data [14-18]. Turkey, an upper-middle-income economy [19], experienced many of these issues in establishing its first active population-based cancer registry.

### Selection of Turkey for the case study

The Pfizer Global Health Partnership (GHP) project provided funding to 31 countries, focusing on innovative cancer - and tobacco control programs [20]. One of the grants was to The New Hope in Health Foundation (SUVAK), a nongovernmental organization working with the Turkish Ministry of Health (MOH), to expand and enhance cancer registries in the provinces of Izmir, Antalya, Samsun, and Erzurum. Another objective was to establish a cancer advocacy network. Funding *began* in 2008, and since these registries were at different stages of their development with varying levels of capacity, funds were used to improve the infrastructure and build the capacity for these cancer registries as needed. The focus was to improve quality control, hire and train additional staff, provide additional training on how to identify and review cases, and proper techniques in re-abstracting. While registries were supported by government funds, money was not available for travel, training and the review process. Pfizer funding was used for these additional costs needed to improve and enhance the registries. While there are numerous registries in Turkey, this case study will focus on the active, population-based registries that were included in the GHP project (Izmir, Antalya, Samsun, and Erzurum). These registries provide an interesting account of the challenges they faced and the lessons learned from which other countries with similar resources can benefit; and it examines the contribution of these registries to the country’s overall cancer control strategy.

## Methods

This research used a qualitative case study approach to detail the process of expanding Turkey’s cancer registries. Data were collected from November 2010-January 2011. Johns Hopkins Institutional Review Board approval was obtained *prior to data collection.*

### Data collected

Qualitative data collection included reviews of the registries’ background documents and online documents from the Turkey’s MOH Cancer Control Department website, organizational surveys, and in-person, in-depth key informant interviews. The documents reviewed provided information related to the formation of the registries and information concerning the registries’ capacity. This background information was used to help in the development of the questions for the key informant interviews and for the organizational survey (described further below). Document review was limited to those appearing in English, from both print and online sources. Additional background information was gathered from individuals outside Turkey with long-standing knowledge of the registries, and this assisted in the development of the topics to be included in interview guides for the key informant interviews [Dr. Brenda Edwards, personal communication; Dr. Joe Harford, personal communication]. Project staff from SUVAK reviewed the interview guides to ensure cultural appropriateness. All interviews were conducted in English; translation for non-English speakers was provided by SUVAK. The key informant interviews (n=10) were conducted in person in Turkey and included SUVAK officials, MOH representatives, registry personnel, and advocacy network representatives.

Finally, an organizational survey was conducted with each registry. Open-ended questions were used to obtain information on organizational structure and capacity, including information on registry personnel; staff skill development; data collection (methods used, data quality assurance methods, and other procedures used to update or improve data collection); data usage (to whom and how disseminated); current funding sources; participation in international activities (meetings and publications); and challenges/barriers faced by the registry.

### Analysis

Interviews were digitally recorded and transcribed. Survey data (open-ended questions) and interview data were content-analyzed using Atlas.ti 5.5 software to identify factors related to registry functioning and progress. Other survey data was categorized and summarized. Representative phrases and quotations from the surveys and key informant interviews were selected to highlight the identified factors. The following factors will be addressed: 1) establishment of the cancer registries in Turkey; 2) organizational structure of the registries; 3) enhancement of the registries; 4) using data to inform policy; and 5) funding and sustainability.

## Results

### Establishment of cancer registries in Turkey

This first registry in Turkey was established in Izmir in 1992. This registry became a member of WHO/International Agency for Research on Cancer’s (IARC) International Association of Cancer Registries in 1995 and the European Network of Cancer Registries (ENCR) in 1997 and was included within the framework of the Middle East Cancer Consortium (MECC) in 2004 [21]. This demonstrates that this registry was meeting international standards with high quality, publishable and representative data [22,23]. Other Turkish provinces worked to establish their cancer registries with Izmir as its model, often with varying degrees of success in being able to produce the necessary level of quality data.

As the registries were established at different times, they have varying levels of capacity. The amount of time the registries have been in existence does reflect on their progress to date, but there are other factors that influence their current success. Table 1 compares basic information on each of the registries in the study that could have such an influence. Location is an especially relevant issue since the registries included in this study represent different areas of the country. Izmir is in the West, Antalya is in the South, Samsun is in the North, and Erzurum is in the East. For example, the Erzurum registry struggled initially and still has not achieved international quality data, but it is deemed important for continued support and funding by MOH. This is largely due to the fact that Eastern Turkey is less developed, has different cancer rates, and these data are needed to provide a comprehensive view of this area of the country. Furthermore, a number of the registries reported lack of skilled staff, and this is especially acute in Erzurum where turnover is higher and the registry has the fewest number of staff due to its lower level of development.

**Table 1:** Comparative look at cancer registries in four provinces of Turkey

**Province(region covered)**	**Year established**	**Local advisory committee**	**Staff capacity**	**Ongoing trainings for staff**	**International quality data**	**International journal publications**	**Specific barriers**
Izmir (Western Turkey)	1992	Yes	32 full time; 10 part time	Yes	Yes	Yes	● Current bureaucratic set-up
							● Missing mortality data
							● Lack of staff
Antalya (Southern Turkey)	1995	Yes	10 full time; 42 part time	Yes	Yes	No	● Missing demographic data
							● Incomplete SEER staging data
Samsun(Northern Turkey)	2001	Yes	16 full time; 12 part time	Yes	No	No	● Some passive data collection methods
							● Missing demographic
Erzurum (Eastern Turkey)	2005	Yes	7 full time; 2 part time	Yes	No	No	● Inadequate access to laboratories
							● Missing demographic data
							● Lack of staff

The Izmir Cancer Registry was the country’s first population-based registry that supported active data collection [21,22]. It was initiated as a collaboration between the University of Massachusetts in the United States and the Ege University of Izmir. At first, the registry lacked the basic infrastructure, but it received both direct and indirect support from numerous international agencies, including the International Agency for Research on Cancer (IARC) and the International Association of Cancer Registries (IACR). Currently, in addition to the Izmir, Antalya, Samsun and Erzurum registries, there are four other functional registries covering 21% of the entire population. Data collected by these registries provide comprehensive understanding of cancer types and patterns that differ by region and are used to formulate Turkey’s cancer projections [24,25].

The interviews indicated that lack of resources is one of the major challenges faced when establishing a registry, but that interaction with cancer registries at the international level helped (Director, Izmir Cancer Registry). For Turkey, getting the Izmir registry to be recognized by international agencies was extremely important. The Izmir Registry was identified as a model program, and this helped lead the way for others in the country. One MOH Official said, “….we had a good model in Izmir Cancer Registry and we tried to apply this model to other provinces we selected”. Thus, with the growing success of the Izmir Registry, the MOH decided to expand the number of active registries in other regions of Turkey to gather more population-based cancer data.

The registry in the southwestern province of Antalya was established next, with the MOH working with such local stakeholders as elected officials and healthcare professionals. Developing a strong working relationship with these stakeholders as well as gaining local commitment and support was a factor identified as contributing to the progress made by the Antalya registry. “There is good ground level support from [the] medical community as well as local leaders such as the mayor”(MOH official).

During subsequent years, additional registries were established, however; several registries failed to succeed. “The main reason of the failure [of a registry] is lack of enthusiasm of the local health authorities about the subject. [There were] problems in the organization of the registries, lack of qualified personnel, lack of the registries’ infrastructure, neglect of both the national and local administrators on the registries..” (MOH official). In 2000, the MOH convened the National Cancer Advisory Board of Turkey to provide overall direction to the country’s cancer registries. With advice from the Board, data were collected only from certain provinces. An MOH official indicated that, “After 2000, we decided to collect data from certain provinces instead of the whole of Turkey because we noticed that it was very difficult… it’s quite impossible to collect reliable information from the whole of [a] country for 70 million people. So we decided to select provinces and collect data”.

### Organizational structure of the registries

**Figure 1:**
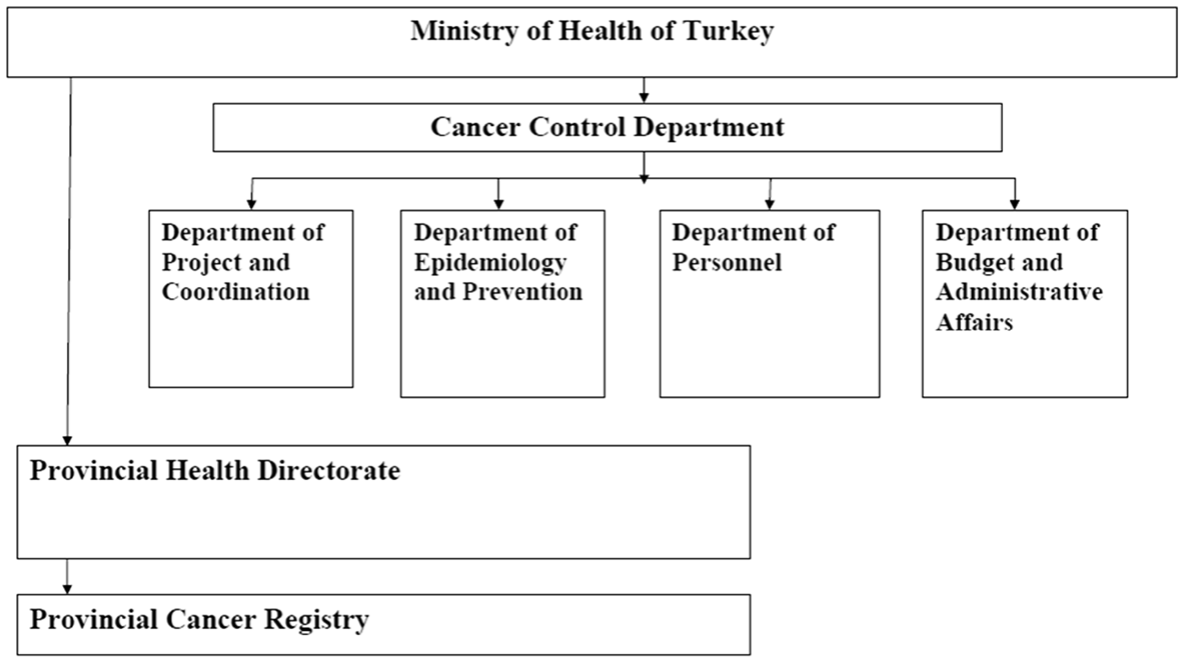
**Administrative oversight of Provincial Cancer Control Registries in Turkey **[24]**.**

The four registries in our case study have a similar structure. The MOH provides oversight of the Provincial Health Directorate or Agency regulating the Provincial Cancer Registry. Thus, the registries are administrated through the Provincial Health Directorate and not at a national level. Figure 1 shows the structural schematic of the Turkish cancer registries. Each registry has a central office operating as the core administrative body gathering data from numerous hospital-based “cancer registry units”. Each registry in Turkey has core staff plus registrars and other full- and part-time personnel. Data are collected from a number of district- level hospitals and other health care facilities. Differences may exist due to differing bureaucratic structures found with in the Provincial Health Directorates.

#### Presence of qualified leadership and personnel

Involved and committed leadership led to the success of the Izmir Cancer Registry and its demonstration that a strong champion from within can advocate with various stakeholders for a registry’s progress. The current Director of the Izmir Cancer Registry -- a physician has been with the project since its initiation and contributed towards establishing successful registries in Turkey. Under her leadership, and with the funding provided by the GHP, the Izmir registry has served as the model for other registries, and staff from Antalya, Samsun, and Erzurum and other registries have trained at Izmir. According to the Director of the Izmir registry, in addition to the staff coming to Izmir for training, Izmir staff themselves travel to the other registries to help them improve their data abstracting and quality control procedures.

Another factor identified is the lack of trained personnel to lead and staff the registries. Having a committed individual in the leadership role is especially important. Lack of leadership is one reason that the Erzurum Cancer Registry has made slower progress. The interviews indicated that not being able to find a medical doctor and finding and training someone only to have them leave are significant and ongoing challenges that are faced. An explanation for this turnover, according to a key informant, was better professional opportunities in other Turkish provinces. In addition to strong leadership, a registry needs qualified staff to function optimally.

##### Enhancement of the registries

Once the registries have the infrastructure in place, ongoing staff training is essential to collect quality data and achieve the international standards set by IARC.

#### Ongoing trainings

Izmir registry was the model and helped provide the training for the other registries *through site visits*. Curriculum included basic training, advanced training, *geographic* mapping (GIS), and CANREG – the software from IARC. *The focus of these site visits was to improve quality control, hire and train additional staff, and provide additional training on how to identify and review cases and proper techniques in re-abstracting.* Since registry personnel need specialized skills to collect comprehensive data and analyze and present accurate results, systematic trainings organized by MOH and SUVAK to establish credentials is necessary. Ongoing meetings where registry personnel discuss how to improve data quality and conduct audits as quality control measures are also training opportunities. Registrars attend international conferences to network with cancer registry experts from other countries and to present their research findings before the international community. While, these opportunities are important to build expertise and skills, they require resources that are not always available.

#### Maintaining quality data

To produce and maintain high-quality data, cancer registries must perform quality assurance activities, as incomplete or missing data will lead to faulty projections and failure to detect patterns. In Samsun, initial audits showed the collected data were not accurate, but further training of registry staff, with technical assistance and site visits from experts, corrected this problem. Teams from other provinces with better success at collecting quality data were also sent by MOH to help. Erzurum has been the focus of some of these efforts, with the result being that clear data trends have begun to emerge and progress is being made. For all the registries, including Izmir, the model registry and ongoing audits are necessary to maintain and enhance data quality.

#### International collaboration and recognition

Over the years, the Izmir Cancer Registry *has achieved success partly because of assistance* from MECC [21]. MECC, with support from the U.S. National Cancer Institute (NCI) of the National Institutes of Health, assists in establishing and enhancing population-based cancer registries in its member countries [26,27]. MECC provides funds and technical assistance to the Izmir Cancer Registry, contributing to its growth and success, with three full-time registrars paid through MECC funds.

Coupled with this, another indicator of Izmir’s success is its international recognition. Data from the Izmir Cancer Registry are included in IARC’s GLOBOCAN project that reports global cancer statistics; GLOBOCAN also cites registry data from Antalya. Data from both these registries are referenced in IARC’s publication, *“Cancer Incidence in Five Continents Vol. IX”,* highlighting again the quality of the data collected by these two registries [28]. Registry personnel in Turkey have published research studies in peer-reviewed international journals, written book chapters, and spoken and presented both *domestically and in other countries, raising their international profile and ensuring continued support from the international community.*

##### Using data to inform policies

Using the quality data collected by the registries, Turkey was able to shape policies and allocate funding and other resources towards preventive measures [29]. For example, lung cancer is the most common type of cancer in males in Turkey [23,25], and in 2009, the MOH took steps towards preventing tobacco consumption by banning smoking in all indoor public places [29].

When the MOH Cancer Control Department presented data obtained from cancer registries as a subset of overarching chronic diseases data, their MOH colleagues recognized and began to respect the cancer registries as models to be replicated for other health projects administered by the Ministry, as illustrated by this quote from a MOH official, *“Cancer statistics became important for the Ministry and even became [a] model for other statistics”.* More importantly, cancer is now firmly in the forefront of the Turkish health agenda. There is now a realization that non-communicable diseases and chronic disease are becoming national priorities *“Turkey finished this fight with acute diseases; we do not have so much maternal deaths, almost 100 percent vaccination rate we now have…It is time for Turkey to fight chronic disease”. (MOH Official).*

##### Funding and sustainability

When the Izmir registry’s data met the international criteria established for quality, it won the support of the MOH. Most of the registries’ funding support comes from the MOH, while the remainder is a mix of international health organizations, corporations, and private philanthropic organizations. With the GHP funds in particular, SUVAK and the MOH were able to train additional registry personnel. Since government funds cannot be used for certain activities such as attending international meetings, the GHP funds defrayed some of the associated costs. As the Izmir and the Antalya registries were already established, these external funds were used to upgrade the infrastructure and conduct audits.

For cancer registries – as for any other program -- to be successful and develop over time, they must demonstrate sustainability once international donors have pulled back. In Turkey, a testament to the credibility and sustainability of the registries is that the MOH now provides the majority of their funding support, as international partners are slowly stepping away. *“[The] Ministry recognized the importance of the registries and provided additional funding and supported setting up of additional of registries” (MOH Official).*

With the government firmly committed to supporting the registries, they will adapt and sustain themselves using existing resources. The MOH also recognizes the importance of building a qualified workforce to support these registries in the longer term. Providing adequate compensation to attract and retain qualified professionals is essential to maintain these registries.

"“If you want to get real benefit from a project…the human resources and manpower is the most important thing for this project, but you have to pay enough money. Otherwise, it is impossible to get qualified persons and human resource” (MOH official)."

Overall functioning of the registries needs to be streamlined for sustainability. As key informants noted, the current multi-layered bureaucratic structure of the cancer registries prevents regular contact between registry staff and the policy-making government agency. Interaction is also limited between the staff and other qualified professionals, which discourages collaboration with the international registry community. Regular interaction between these various stakeholders can lead to more efficient and effective registry operation.

##### Creation of an advocacy network

A unique feature that may contribute to long-term registry success could be the creation of an advocacy network in Turkey. SUVAK brought a number of disparate organizations under a single platform called “Hand in Hand Against Cancer”, and, with GHP funding support, organized a capacity-building training series for these NGOs to empower and unify them towards a shared vision. The key informants indicated that,*“…in Turkey we don’t have enough NGO activities, so we are very hungry for that kind of project”.* The network has developed resources to build the public’s cancer awareness and educate them. More importantly, it has given a voice to those most affected by cancer and has presented *their work* to the Turkish Parliament. The network has the capacity to translate data collected by the registries for advocacy purposes and to push cancer into the country’s mainstream health agenda.

## Discussion

This study illustrates the trials and tribulations of Turkey as it established cancer registries and expanded their cancer control program. It outlines the real life struggles the registry workforce and the ministry officials faced at the initial stages and how they were overcome. It provides a summary of the difficulties that can potentially be avoided by other countries with limited resources. *The registries we highlight in this case study found that location (and thus level of development), proper staffing, and varying levels of capacity were challenges that needed to be addressed to bring other registries up to par. But with sufficient funding and support from international organizations, the registries were able to overcome these barriers, establish successful registries, and gain recognition of the importance of cancer surveillance by the MOH.*

For LMICs establishing a population-based registry, having government-issued policies in place could be beneficial, as in Turkey [30,31]. Health care professionals are more likely to respond to government-mandated directives on data reporting than to requests from the registries themselves. Key factors for gaining wider support and acceptance include demonstrating: 1) the government’s support through the presence of such policies or directives, and 2) the value of the data collected by the cancer registries. *In this study, convincing the MOH was the first step to making this happen. But with Turkey’s success as a role model for other countries, this could perhaps be achieved more quickly for other countries.*

Turkey faced many challenges in establishing and expanding its cancer registries. Its lessons are useful for other countries with limited resources to begin developing effective registries. According to key informants, setting up registries was time-intensive, but often certain stakeholders wanted a new registry created quickly to collect quality data and function efficiently. Educating officials on the importance of building the capacity and technical skills of the registry staff -- a process which, according to IARC, takes several years -- is an important initial step. *This is clear in the case of Turkey, and could be used as an example of the often-lengthy process for other countries that expect too much in too short a time.*

Informant interviews also revealed how physicians and other potential registry workforce perceived cancer registrars. Initially, it was difficult to attract qualified candidates to this field, as it was undervalued as a career choice. However, with growing national and international recognition, that perception is changing; the MOH and other stakeholders understand the value of building a qualified workforce and providing adequate compensation. *Again, these lessons learned in Turkey could serve as guiding principles for other countries looking to establish their own registries.*

The provincial cancer registries in Turkey are currently administered by a multi-layered bureaucratic structure that prevents free interaction between registry staff, researchers from outside organizations, and policy makers. Key informants recommended moving the registries under the umbrella of a national research institution to circumvent some of the bureaucracy; this could also allow registry personnel to operate more independently. *This is a lesson learned that could benefit countries who are looking to establish registries in the near future.*

It should be noted that currently a limitation faced by Turkey is that mortality data is not collected, and this restricts the comprehensiveness of its registry data. In accordance with international standards, Turkey is implementing new legislation requiring all family physicians to complete a cause-of-death form that includes cancer mortality data. The MOH also plans to collect cancer survival data*. It is recommended that countries that use Turkey as a model for establishing cancer registries take this limitation into account when establishing their own mechanisms.*

## Conclusion

This study demonstrates how Turkey used resources from multiple sources to enhance its population-based cancer registry system in four provinces. With renewed international interest in non-communicable diseases and cancer following the 2011 UN high-level meeting on NCDs, low- and middle- income countries can benefit from Turkey’s experience. Other countries can utilize lessons learned from Turkey as they address cancer burden within their own borders and establish their own registries, learning from each other to create a world that has a better handle on this serious threat to global health.

## Competing interests

The authors declare that they have no competing interests.

## Authors' contributions

FS designed and conceptualized the case study, collected the data, helped in interpretation of the data, provided the structure for the paper, and prepared the final draft of the paper. MK –helped with the data analysis, helped with the interpretation of the data and writing of the manuscript. NKconducted the analysis and wrote the 1^st^ draft of the paper. SE- provided data for the case study, helped in interpretation of the findings, and reviewed and contributed to the writing of the paper. MS helped in designing the data collection instruments, helped with the data collection, and contributed to writing and reviewing the paper. YP- assisted with data collection and review and contributed to the content of the paper. All authors read and approved the final manuscript.
